# The obesity-related chronic disease index (ORCDi), a novel composite metric to quantify ORCD burden: an ecological study across U.S. census tracts

**DOI:** 10.1016/j.lana.2026.101547

**Published:** 2026-07-01

**Authors:** Md Roungu Ahmmad, Fazlay Faruque, Xiaoli Zhang

**Affiliations:** a USF-Health, College of Nursing, University of South Florida, Tampa, FL, USA; b Geospatial Health Research Lab, Department of Preventive Medicine, University of Mississippi Medical Center, Jackson, MS, USA; c Biostatistics Core, USF Health, College of Nursing, University of South Florida, Tampa, FL, USA; d The Chi-Square Research Lab, Biostatistics Core, USF Health, College of Nursing, University of South Florida, Tampa, FL, USA

**Keywords:** Obesity-related multimorbidity, Social vulnerability, Precision public health, Principal component analysis, ORCD

## Abstract

**Background:**

Obesity contributes substantially to chronic disease multimorbidity in the U.S., yet no composite metric exists to quantify the cumulative burden of obesity-related chronic diseases at the community level. We aimed to develop and validate the obesity-related chronic disease index (ORCDi) and examine its spatial distribution and associations with social vulnerability domains.

**Methods:**

We used Centers for Disease Control and Prevention (CDC) PLACES 2024 estimates for nine obesity-related conditions across 83,522 U.S. census-tracts. Principal component analysis was applied to derive the ORCDi, and chronic disease properties were assessed using Cronbach's α, Guttman's λ6, item–total correlations, Bartlett's test, and Kaiser-Meyer-Olkin (KMO) adequacy. Convergent validity was evaluated using regression models against social vulnerability indices and rural-urban commuting area (RUCA). Spatial dependence and clustering or hotspots were assessed using Global Moran's I and Local Indicators of Spatial Association (LISA).

**Findings:**

The ORCDi demonstrated internal consistency (Cronbach's α = 0.94; Guttman's λ6 = 0.97) and a coherent factor structure (KMO = 0.81; Bartlett's χ^2^ = 1068,915; p < 0.0001). The first principal component accounted for 70.52% of the total variance and exhibited significant factor loadings for chronic obstructive pulmonary disease (0.94), stroke (0.94), high blood pressure (0.93), coronary heart disease (0.91), and diabetes (0.89), supporting a shared multimorbidity dimension underlying the index. Convergent validity was supported by moderate associations with socioeconomic vulnerability (β = 15.02; R^2^ = 0.19), age and disability (β = 15.74; R^2^ = 0.21), and rurality (β = 1.42; R^2^ = 0.14). Spatial analysis demonstrated notable geographic clustering (Moran's I = 0.71; p < 0.0001), with high–high ORCDi concentrations in the Deep South, Mississippi Delta, Appalachia, and parts of Texas and Oklahoma.

**Interpretation:**

The ORCDi is a composite measure of obesity-related chronic disease burden at the community level. The index demonstrated significant internal consistency, substantial geographic variation, and meaningful associations with socioeconomic disadvantage and rural health disparities. Future studies should evaluate its performance in independent datasets and assess its utility for public health surveillance and resource allocation.

**Funding:**

The study was not supported by external funding.

**Research in context**

**Evidence before this study**

Before undertaking this study, we conducted a literature search in google scholar, PubMed, and Web of Science for articles published from database inception to Nov 30, 2025, without language restrictions. We used combinations of the terms “composite index”, “obesity-related chronic disease”, “chronic disease burden”, “health index”, “vulnerability index”, “social vulnerability index”, “area deprivation index”, and “spatial epidemiology”. We reviewed studies that developed or applied population-level indices to characterize disease burden, multimorbidity, health vulnerability, or geographic health disparities. Existing indices, including the Social Vulnerability Index, Area Deprivation Index, and Rural-Urban Commuting Area classifications, were designed primarily to capture social, economic, or geographic vulnerability rather than the cumulative burden of obesity-related chronic diseases. Although some studies have investigated obesity-related conditions and multimorbidity separately, we found no validated national index specifically developed to quantify obesity-related chronic disease at the U.S. census-tract level. Furthermore, evidence describing the spatial distribution and clustering of obesity-related chronic disease across all U.S. census tracts remains limited.

**Added value of this study**

To our knowledge, this study is the first to develop and validate a census tract-level obesity-related chronic disease index (ORCDi) for the U.S., based on principal component analysis of nine obesity-associated chronic conditions measured across 83,522 census tracts in the CDC PLACES dataset. The index demonstrated reliability and structural validity and showed meaningful associations with established measures of social vulnerability and rurality. By integrating geospatial analyses, this study provides a standardized and robust approach for quantifying community-level obesity-related multimorbidity burden across the U.S. The findings revealed substantial geographic inequities, with the highest burdens concentrated in the Deep South, Mississippi Delta, Appalachia, and parts of Texas and Oklahoma.

**Implications of all the available evidence**

The ORCDi provides a novel tool for quantifying obesity-related chronic disease burden at the community level and complements existing measures of social and geographic vulnerability. Its application may improve public health surveillance, facilitate the identification of high-burden communities, and inform geographically targeted prevention, resource allocation, and chronic disease management strategies. Future research should assess the external validity and broader applicability of the ORCDi across populations, geographic settings, and health systems to support its use in public health practice and policy.

## Introduction

Obesity is a major public health challenge and a leading cause for a wide range of chronic diseases, including diabetes, hypertension, heart disease, stroke, asthma, arthritis, and chronic obstructive pulmonary disease (COPD).^1^ These conditions frequently co-occur and interact, resulting in compounded health burden, increased disability and mortality–particularly among socioeconomically disadvantaged and medically underserved populations.^1^^,^^2^ While population health surveillance often reports disease prevalence individually, obesity-related chronic diseases (ORCD) tend to cluster and share biological risk trails, contributing to multimorbidity and reduced quality of life.^3^ However, there is currently no standardized index that captures the combined burden of ORCD at small geographic unites, such as census tracts or community-levels, where local health disparities are most visible and relevant for intervention.

ORCD are biologically and socioeconomically interconnected, often sharing common metabolic, inflammatory, and lifestyle–driven pathways such as insulin resistance, systemic inflammation, and physical inactivity.^4^ These shared mechanisms lead to the progressive development of multiple chronic co-occurring multimorbidity conditions rather than isolated illnesses.^5^ The clustering of these conditions, often reflected by social vulnerability index (SVI) and rural-urban commuting area (RUCA) amplifies health risks, accelerates disability, and worsens quality of life.^6^ Therefore, a composite index is essential to capture the cumulative and synergistic burden at the community-levels. This composite approach provides a more comprehensive and realistic representation of population-level health burden, supporting better surveillance, geospatial clustering, and policy prioritization.^7^

Growing evidence shows ORCD are unevenly distributed across U.S. communities, with persistent spatial disparities.^8^ Composite health indices and multilevel surveillance frameworks have been increasingly used to quantify population-level disease burden and guide targeted prevention strategies.^8^^,^^9^ Methodological advancements in index construction have improved the capacity to assess co-occurring chronic disease using reliable composite indicators.^10^ However, ongoing debates remain regarding the conceptualization, validity, and interpretation of population health indices, particularly for chronic disease influenced by social and geographic contexts.^11^ Recent international applications underscore the potential of spatially evaluated indices to reveal hidden regional disparities and inform health policy.^8^^,^^11^ Together, this literature supports the need for robust, spatially explicit, composite chronic-disease assessments. Existing indices, such as SVI and the Area Deprivation Index, effectively capture socioeconomic vulnerability and area-level disadvantages.^12^^,^^13^ However, these tools do not reflect the interconnected disease burden arising specifically from obesity-related chronic disease, nor do they quantify spatial patterns of obesity-related health burden.

To address this critical gap, this study aimed to introduce a novel ORCD index (ORCDi) that integrates nine high-impact obesity-related chronic diseases at the census tracts-level. Unlike existing tools that profile single disease prevalence or broader socioeconomic vulnerability, the ORCDi is specifically designed to capture the cumulative, obesity-related burden of multimorbidity across communities in a quantifiable and comparable way. This index examines the relationships between obesity-related disease burden, social vulnerability, and rural-urban disparities to identify geographic patterns and spatial clustering of health vulnerability. The resulting score is easy-to-interpret, enabling data-driven decision-making for resource allocation, intervention prioritization, and evidence-based policy planning. Ultimately, this index provides a clearer picture of overall community health and helps identify areas most in need of targeted support in the U.S.

## Methods

### Study design and framework

The ORCDi was derived using principal component analysis (PCA) of nine ORCD prevalence indicators from CDC PLACES data.^14^ Census tracts were used as the primary unit of analysis, offering a relatively stable fine-grained spatial perspective on community-levels health vulnerability.^15^

### Data sources

#### CDC PLACES data

The CDC PLACES Project, formerly known as the 500 Cities Project, provides model-based, small-area estimates for chronic disease prevalence, derived from the 2024 pooled modeling cycle (released in 2025) which is primarily based on Behavioral Risk Factor Surveillance System (BRFSS) 2022 data.^14^ We extracted age-adjusted prevalence (%) data for nine ORCD conditions: obesity, diabetes, high blood pressure, coronary heart disease, stroke, COPD, asthma, arthritis, and high cholesterol. Prevalence estimates were obtained at the census tracts level using multilevel regression and post-stratification applied to BRFSS data.^14^ These estimates were modeled rather than directly observed, particularly in sparsely populated rural tracts and should not be interpreted as direct tracts-level clinical measurement. Obesity was defined as body mass index (BMI) ≥30 kg/m^2^ diabetes, high blood pressure, coronary heart disease, stroke, COPD, asthma, arthritis, and high cholesterol were defined based on self-reported clinician diagnosis. These diseases were selected based on their established biological and epidemiological associations.^16^ The detailed definition was provided in the supplemental section, the chronic disease definition.

#### USDA RUCA data

The RUCA codes were sourced from the U.S. Department of Agriculture (USDA)'s Economic Research Service (ERS) to characterize population density, commuting interactions, and rural-urban gradients at the census tracts level.^17^ RUCA codes range from 1 to 10, reflecting the degree of rurality, where RUCA 1 classified as metropolitan core and RUCA 10 classified as most rural and isolated areas. These classifications were used to examine rural-urban gradients for the ORCDi and comparative vulnerability pattens.^15^^,^^18^

#### CDC/ATSDR SVI data

The CDC/Agency for Toxic Substances and Disease Registry (ATSDR) SVI was used to assess convergent construct validity for the ORCDi, as it captures census tracts level socioeconomic, demographic, and housing-related vulnerabilities.^19^ The SVI quantifies community-levels vulnerability into four thematic domains: (Theme 1) Socioeconomic Status (e.g., poverty, unemployment, income, education); (Theme 2) Household Composition & Disability (e.g., age ≥65 years, age ≤17 years, disability status, single-parent households); (Theme 3) Minority Status and Language (e.g., racial/ethnic minority populations and limited English proficiency); and (Theme 4) Housing Type and Transportation (e.g., multi-unit structures, crowding, vehicle access, group quarters). Domain and overall SVI range from 0 to 1, with higher values indicating greater social vulnerability.^20^

### Variable selection and conceptual framework

The nine chronic disease indicators included in the analysis based on well-characterized ORCD relationships documented in previous studies.^5^^,^^6^ These conditions are well-known to co-occur in metabolic, pulmonary, cardiovascular, and inflammatory clusters, contributing to multimorbidity.^21^ The conceptual model assumed ORCD as a composite affecting multiple chronic disease pathways simultaneously.^22^

### ORCDi derivation

All prevalences were standardized using z-score normalization to address scale differences of different variables and allowed unbiased principal component analysis (PCA) computation.^23^ Suppose i=1,…,N denoted census tracts and j=1,…,9 denoted the chronic disease indicators. Each indicator $X_{ij\;}$ represented prevalence (%) of condition j in tract i. A normalized z-score is derived by $Z_{ij}=\frac{X_{ij}\;-\;{\overset{ˉ}{X}}_{j,}}{s_{j}}$, where ${\overset{ˉ}{X}}_{j\;}$ and $s_{j}$ were the mean and standard deviation of variable j across all census tracts. The ORCDi was derived using these $Z_{ij}$ score based on PCA where, PCA applied standardized prevalence values to identify latent constructs. PCA was performed on the correlation matrix: $R=\frac{1}{n\;-\;1}Z^{⊤}Z$, where Z was the n×p matrix of standardized observations. The correlation matrix was decomposed as: $R=V\Lambda V^{⊤}$, where $\Lambda =\text{diag}\left(\lambda_{1},\lambda_{2},\ldots ,\lambda_{p}\right)$ contains eigenvalues ($\lambda_{1}\geq \lambda_{2}\geq \cdots \geq \lambda_{p}$), $V=\left(v_{1},v_{2},\ldots ,v_{p}\right)$ contains the corresponding eigenvectors. The k-th principal component was defined as ${\text{PC}}_{k}=v_{k1}Z_{1}+v_{k2}Z_{2}+\cdots +v_{kp}Z_{p}$, where $v_{kj}$ was the loading of variable j on component k. The proportion of variance explained by component k is $\text{Var}\left({\text{PC}}_{k}\right)=\frac{\lambda_{k}}{\sum_{j\;=\;1}^{p}\lambda_{j}}$.

Component retention was based on the Kaiser criterion (eigenvalue >1), proportion of explained variance (>60%). Factor loadings (≥0.70) and contribution scores were examined to determine variable importance in index construction.^24^ Since only the first principal component represented a coherent underlying structure with explanatory power, it was retained to derive the ORCDi composite score.

$${\text{ORCDi}}_{i}^{\text{raw}}=\sum_{j\;=\;1}^{9}w_{1j}Z_{ij}\text{,}$$

where, $w_{1j}$ is the PC1 loading for indicator j, $Z_{ij}$ is the standardized Prevalence of indicator j in census tracts i. This raw index reflected the magnitude of obesity-related multimorbidity in continuous form but was not yet bound for interpretation. To improve interpretability and allow meaningful classification, the scores were min–max rescaled to a 0–100 range using:

$${\text{ORCDi}}_{i}=100\;\times \frac{{\text{ORCDi}}_{i}^{\text{raw}}\;-\;\underset{k}{\min}\left({\text{ORCDi}}_{k}^{\text{raw}}\right)}{\underset{k}{\max}\left({\text{ORCDi}}_{k}^{\text{raw}}\right)\;-\;\underset{k}{\min}\left({\text{ORCDi}}_{k}^{\text{raw}}\right)}\text{,}$$

this transformation preserved distributional properties while enhancing interpretability, where higher ORCDi indicated greater ORCD burden and vulnerability.

To facilitate interpretation and visualization, census tracts were categorized into low, moderate, and high vulnerability groups using k-means clustering applied to the ORCDi. The clustering algorithm partitioned tracts into three mutually exclusive groups based on similarity in index values, minimizing within-group variance while maximizing between-group separation. These categories were used for descriptive mapping, spatial clustering, hotspots identification and policy implication.

### Missing data handling

Missing values were limited to high blood pressure and high cholesterol prevalence estimates, affecting 5007 of 83,522 census tracts (6.0%) for each variable. Sensitivity analyses based on complete-case data produced findings that were highly consistent with those from the primary imputed dataset, indicating minimal influence of the imputation procedure on index derivation (Supplementary Table S1). To preserve complete geographic coverage and avoid exclusion of census tracts from index derivation, missing values were replaced using the mean of the corresponding non-missing prevalence. Distributional assessments indicated prevalence patterns were comparable before and after imputation. Sensitivity analyses were conducted for complete cases to demonstrate consistent with the primary findings.

### Reliability and validity testing

#### Internal consistency and reliability

The internal consistency of the ORCDi was assessed using Cronbach's alpha (α), standardized alpha, Guttman's Lambda-6 (λ6), and McDonald's Omega (ω), which evaluate the degree of shared variance among the 9 chronic disease indicators.^25^^,^^26^ Item-total correlations were computed to assess individual item contributions to the overall index, where values > 0.30 indicated adequate discrimination.^27^ Average and median inter-item correlations were used to evaluate construct cohesiveness, with values between 0.30 and 0.70 being considered optimal for multidimensional health-related scales.^27^ The signal-to-noise ratio and variance of inter-item correlations were used to assess measurement stability and precision.^25^

The suitability of the correlation matrix for dimensionality reduction was evaluated using the KMO test, which measures sampling adequacy, where values > 0.70 indicate excellent factorability.^28^ Bartlett's Test of Sphericity was used to assess whether the correlation matrix differed significantly from an identity matrix.^29^ A significant result (p < 0.05) established sufficient intercorrelation among indicators to justify PCA. Structural stability was assessed using Jennrich's test, which compares correlation matrices between training and testing subsets to evaluate whether the inter-item relationships were invariant across samples. A non-significant result supports structural consistency, confirming that the component structure was reproducible.^30^ Before conducting Jennrich's test and correlation heatmaps, the dataset was randomly partitioned into a 70% training and a 30% testing set. The training set was used to estimate the model, while the testing set was used for internal validation and comparison. Further, convergent validity of the ORCDi was assessed by examining its associations with established contextual vulnerability measures, including SVI domains and RUCA, using linear regression models to evaluate the strength and consistency of these relationships.

Finally, ORCDi distributions were compared across levels of rurality, providing evidence that the index captures expected geographic variation in chronic disease burden. To assess spatial validity, we quantified spatial autocorrelation of ORCDi across census tracts using Global Moran's I and identified localized significant clusters or to identified the hotspots using Local Indicators of Spatial Association (LISA). The presence of significant clustering indicated non-random geographic patterning consistent with established regional disparities in chronic disease burden. All analyses were conducted in R (version 4.5.1).

### Ethical approval

This study used publicly available, de-identified data from the CDC PLACES Project, the ATSDR SVI, and the USDA RUCA classifications. Because the study involved secondary analysis of publicly available, de-identified data and did not involve human participants, informed consent was not required, and the study was exempt from institutional review board oversight in accordance with applicable federal regulations.

### Role of the funding source

The study was not supported by external funding. Startup funds from the University of South Florida supported publication-related expenses. The funding source had no role in the study design, data acquisition, analysis, interpretation, manuscript preparation, or the decision to submit the manuscript for publication.

## Results

A total of 83,522 U.S. census tracts were included in the analysis (Table 1). The prevalence of obesity, diabetes, high blood pressure, coronary heart disease, stroke, COPD, and arthritis varied substantially across census tracts. Mean (SD) prevalence estimates were 34.4% (8.0) for obesity, 12.4% (5.1) for diabetes, 33.8% (10.4) for high blood pressure, 7.0% (3.6) for coronary heart disease, and 3.7% (1.8) for stroke. The observed ranges were 10.4–64.4% for obesity, 0.7–45.7% for diabetes, 4.1–80.2% for high blood pressure, 0.4–37.1% for coronary heart disease, and 0.2–22.6% for stroke, highlighting substantial geographic variability across communities. The overall KMO value was 0.81, reflecting good sampling adequacy for factor analysis. Item-level KMO scores ranged from 0.73 to 0.90, demonstrating a significant adequacy. Bartlett's test of sphericity was statistically significant (χ^2^ = 1,068,915; df = 36; p < 0.001), confirming that inter-item correlations were sufficiently large for dimensionality reduction with coherent and correlated data structure suitable for deriving a composite index.

**Table 1:** Descriptive statistics of obesity-related chronic disease indicators across census tracts and Kaiser-Meyer-Olkin (KMO) measure of sampling adequacy and Bartlett's Test of Sphericity with Jennrich Test for consistency and stability across samples (N = 83,522).

Characteristic	Mean (SD) [Min, Max] total CT, N = 83,522	KMO measures
Stroke	3.7 (1.3) [0.2, 22.6]	0.79
Arthritis	27.2 (6.8) [2.6, 59.1]	0.82
Asthma	10.6 (1.4) [5.0, 20.7]	0.73
Obesity	34.4 (7.2) [10.4, 64.4]	0.77
COPD	7.5 (2.9) [0.7, 34.9]	0.90
Coronary heart disease	7.0 (2.2) [0.4, 37.1]	0.78
High blood pressure	33.8 (7.2) [4.1, 80.2]	0.84
Diabetes	12.4 (3.8) [0.7, 45.7]	0.80
High cholesterol	34.9 (4.5) [9.0, 58.9]	0.79
Bartlett's Test	Overall MSA = 0.81, χ^2^ = 1,068,915, df = 36, p < 0.0001	
Jennrich Test	χ^2^ = - 262.01, p > 0.9	

**Notes.** MSA, Measure of Sampling Adequacy; χ^2^, chi-square; df, degrees of freedom; p, p-value.

**Fig. 1:**
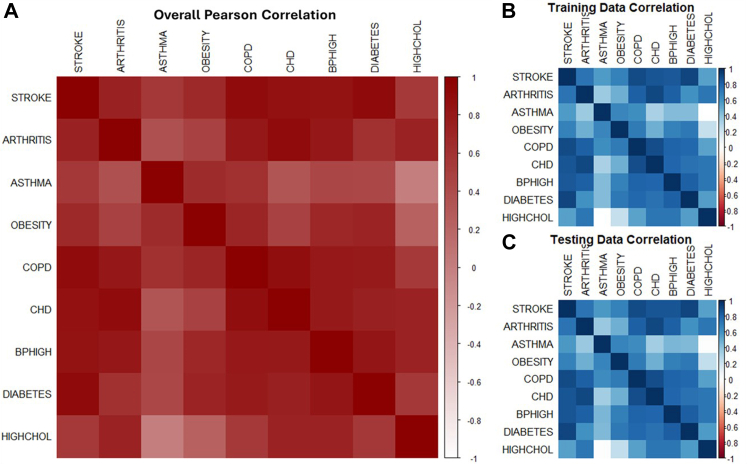
Pearson correlation heatmaps of standardized obesity-related chronic disease prevalence across U.S. census tracts. A) Full dataset overall correlation matrix; B) Training-set correlation matrix (70% random sample); C) Testing-set correlation matrix (30% random sample). Pairwise Pearson correlation coefficients were computed using complete observations to quantify shared variance among the ten standardized ORCD components. Color intensity reflects the magnitude and direction of correlations, with warmer tones indicating stronger positive associations. COPD, Chronic obstructive pulmonary disease; CHD, Coronary heart disease; BPHIGH, High blood pressure; HIGHCOL, High cholesterol.

The correlation heatmap showed moderate to higher positive correlations, with the highest correlation observed between diabetes, high blood pressure, coronary heart disease, COPD and obesity, reflecting a significant association among cardiometabolic and inflammatory conditions (Fig. 1A). Correlation of training and testing data showed nearly identical patterns across census tracts (Fig. 1B and C). Jennrich test (χ^2^ = −226.01, p > 0.9) showed that the strength and direction of correlations were consistent across training and testing samples, indicating stable interrelationships among indicators and supporting the structural consistency of the composite scores.

**Fig. 2:**
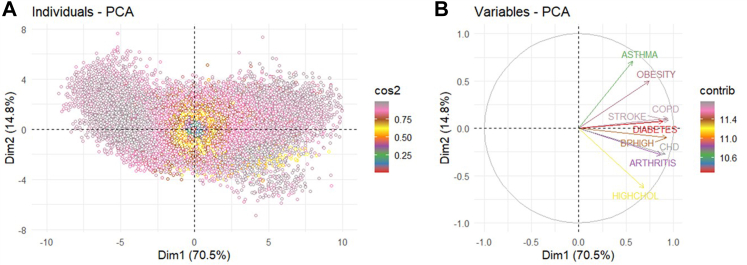
Principal component analysis (PCA) of obesity-related chronic disease burden across U.S. census tracts. (A) Individuals-PCA (at individual tract level): Scatter plot of each individual census tract projected onto the first two principal components. Dim 1 explains 70.5% of the total variance, while Dim 2 explains 14.8% and reflects secondary variation. Point color indicates the quality of representation on the component space. (B) Variables-PCA (at each variable level): Correlation circle showing loadings of chronic disease indicators on the first two principal components. Arrow length reflects contribution magnitude, and direction indicates correlation structure among indicators. Dim 1 captures the primary construct underlying the obesity-related chronic disease index (ORCDi). COPD, Chronic obstructive pulmonary disease; CHD, Coronary heart disease; BPHIGH, High blood pressure; HIGHCOL, High cholesterol.

The first principal component accounted for the largest proportion of variance, whereas the remaining components contributed substantially less (Fig. 2; Supplementary Table S2). The PC1 accounted for 70.5% of the total variance with an eigenvalue of 6.35, capturing most of the shared information. The PC2 explained an additional 14.8%, while the other PCs each accounted for less than 6% of the variance. PC1 demonstrated uniformly significant positive loadings including COPD (0.94), stroke (0.94), high blood pressure (0.93), coronary heart disease (0.91), diabetes (0.89), and arthritis (0.87), representing a shared latent construct of ORCD burden. Obesity had a significant loading on PC1 (0.74). The contributions of each individual chronic disease and each variable on PC1 and PC2 were also explained by the biplot and correlation circle plot (Fig. 2A and B). The first component (Dim 1, 70.5%) represents a dominant cardiometabolic-vascular dimension, characterized by significant positive loadings from COPD, stroke, high blood pressure, coronary heart disease, diabetes, and arthritis. The alignment and similar direction of these vectors indicated substantial shared variance and clustering. The second component (Dim 2, 14.8% explained variance) captures secondary variation, driven primarily by asthma, which loads distinctly along this axis. Arrow length reflects relative contribution magnitude, while direction indicates correlation structure across components.

Internal consistency of ORCDi derivation were further tested by overall Cronbach's alpha and other metrics (Table 2). The overall Cronbach's alpha was 0.94 with a range of 0.93–0.95 if item is deleted. Guttman's λ6 is 0.97 with a narrow range of 0.97–0.98 for each item. The average mean and median inter-item correlations are 0.65 (range: 0.62–0.72) and 0.71 (range: 0.66–0.73) respectively, demonstrating high stability and precision across components, whereas asthma and high cholesterol showed the highest median inter-item correlation of 0.73. The high signal-to-noise ratio of 20.08 and minimal alpha standard error of 0.01 confirm measurement precision and stable reliability. Item-total correlation analyses showed cohesion across most indicators, with the average of item–total correlations of 0.83. COPD, high blood pressure, coronary heart disease, and stroke exhibited the highest corrected item–total correlations (0.88–0.94). obesity, diabetes, and arthritis also demonstrated substantial contributions, followed by asthma and high cholesterol. The average discrimination indices was approximately 80%, reinforcing distinct item differentiation and the central role of cardiometabolic and inflammatory conditions in anchoring the ORCDi.

**Table 2:** Internal consistency and reliability statistics of the ORCDi.

Indicator	Item-level reliability metrics						Item–total correlation and discrimination		
	Cronbach α if item deleted	Guttman λ6	Average inter-item correlation	Median inter-item correlation	Signal-to-noise ratio	Variance of Inter-item correlations	Item–total correlation	Corrected item–total	Discrimination index (r.drop)
Stroke	0.93	0.97	0.62	0.69	13.16	0.04	0.94	0.94	0.92
Arthritis	0.94	0.97	0.64	0.69	14.4	0.05	0.86	0.86	0.82
Asthma	0.95	0.98	0.72	0.73	20.08	0.02	0.59	0.56	0.49
Obesity	0.94	0.98	0.67	0.73	16.36	0.05	0.75	0.73	0.69
COPD	0.93	0.97	0.62	0.68	13.14	0.05	0.94	0.94	0.92
Coronary heart disease	0.93	0.97	0.63	0.68	13.78	0.04	0.9	0.9	0.87
High blood pressure	0.93	0.97	0.63	0.66	13.36	0.05	0.93	0.92	0.9
Diabetes	0.93	0.97	0.64	0.69	14.09	0.05	0.88	0.88	0.85
High cholesterol	0.95	0.98	0.69	0.73	18.01	0.03	0.68	0.65	0.59
Overall reliability metric	0.94	0.97	0.65	0.71	16.81	0.01	0.83	0.82	0.78

**Notes:** Cronbach's α if item deleted indicates the internal consistency of the scale if a given indicator were removed. Guttman's λ6 represents a lower-bound estimate of reliability. The average and median inter-item correlations quantify the degree of shared variance across indicators. The signal-to-noise ratio reflects the ratio of true score variance to error variance. Variance of inter-item correlations indicates homogeneity of item relationships. Item-total and corrected item–total correlations measure the strength of association between each indicator and the composite scale. The discrimination index (r.drop) reflects the contribution of each item to differentiating between high and low index scores.

Item-level reliability metrics and item–total correlation and discrimination metrics.

**Fig. 3:**
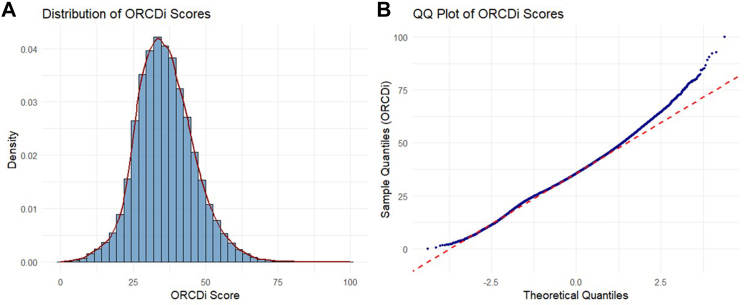
Distribution and normality assessment of the obesity-related chronic disease index (ORCDi) across U.S. census tracts. (A) Histogram and kernel density curve illustrating the empirical distribution of the ORCDi across 83,522 census tracts. (B) Quantile–quantile (QQ) plot comparing sample quantiles with normal theoretical quantiles. Deviations from the 45-degree reference line at the upper tail indicate mild right-skewness, reflecting higher-burden census tracts.

PC1 was used to construct the ORCDi because it explained the highest variance. The ORCDi ranged from 0.00 to 100.00, with a median of 35.87 and a mean of 36.52. The distribution of ORCDi appeared to be unimodal and approximately symmetric, with most census tracts scoring between 25 and 50 (Fig. 3A). The interquartile range (29.59–42.80) shows moderate variability, with most census tracts exhibiting low to moderate ORCD burden, while a smaller subset reached substantially higher scores, reflecting concentrated high-risk clustering. The Q–Q plot demonstrates that the central portion of the ORCDi distribution aligns closely with the theoretical normal line (Fig. 3B). However, slight deviations were observed at both tails with right tail showing more deviations. Both tails showed higher-than-expected observed values. These deviations implied mild skewness and the presence of tracts with disproportionately high disease burden.

**Fig. 4:**
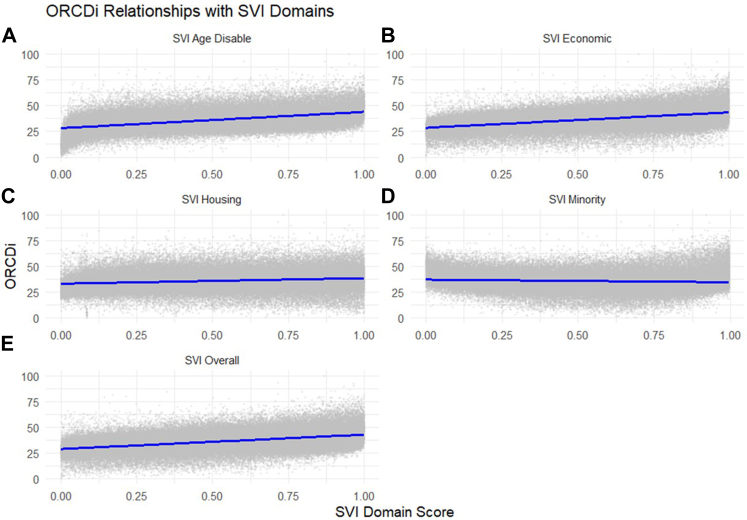
Convergence validity assessment of the obesity-related chronic disease index (ORCDi) using social vulnerability index (SVI) domains. (A–E) Scatterplots illustrate relationships between ORCDi scores and SVI domains (socioeconomic status, age/disability, housing/transportation, and minority status) and the overall SVI index across census tracts. Trend lines indicate the direction of association between contextual vulnerability and obesity-related chronic disease burden.

Next, we examined the convergence of the ORCDi by testing its associations with overall SVI index as well as its four domains (Fig. 4). ORCDi demonstrated positive linear associations with age and disability, socioeconomic status, housing and transportation, and the overall SVI score, indicating higher obesity-related chronic disease burden in census tracts characterized by greater structural and demographic vulnerability. The highest gradients were observed for the age and disability and socioeconomic domains, consistent with elevated chronic disease burden in areas with aging populations and socioeconomic disadvantage. In contrast, the minority status domain exhibited a weak and slightly inverse relationship with ORCDi, indicating that racial/ethnic composition alone does not parallel the spatial clustering of multimorbidity at the census tracts level.

**Fig. 5:**
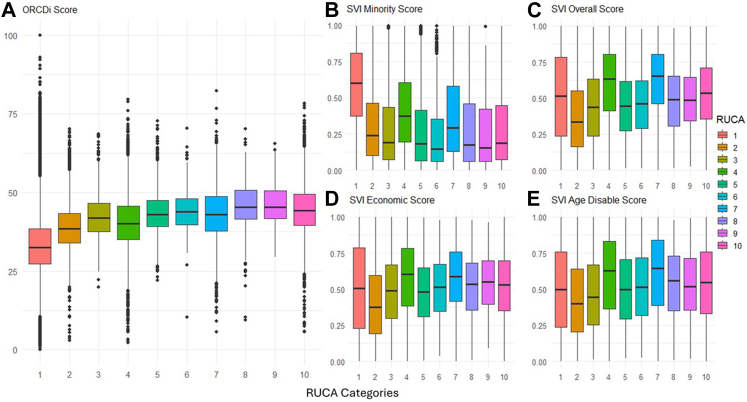
Rural-urban distribution of the obesity-related chronic disease index (ORCDi) and social vulnerability index (SVI) scores across U.S. census tracts. (A) Boxplots of ORCDi scores across Rural-urban commuting area (RUCA) categories (1 = most urban to 10 = most rural). (B–E) Corresponding distributions of SVI minority status, Overall SVI, socioeconomic status, and household composition & disability domain scores across RUCA classifications.

We further examined the distribution of ORCDi across RUCA, where increasing RUCA values representing the degree of rurality (Fig. 5). As shown in Fig. 5A, ORCDi increased progressively across RUCA, with higher median values observed in more rural tracts (RUCA 8–10). Parallel rural–urban gradients were observed for the SVI overall score (Fig. 5C), as well as the socioeconomic (Fig. 5D) and age/disability (Fig. 5E) domains, all of which demonstrated elevated vulnerability in nonmetropolitan classifications. Although the SVI minority status domain (Fig. 5B) exhibited greater dispersion and a less strict monotonic pattern, its distribution remained broadly structured by RUCA level. These associations were demonstrated in linear regression models (Table 3). For example, a one-unit increase in SVI socioeconomic score was associated with a 15.02-point increase in ORCDi, while each one-category increase in RUCA corresponded to a 1.42-point increase. Model fit statistics were consistent with the correlation patterns. The linear regression yielded consistent directional results, aligning with key structural and geographic determinants of community-level disease vulnerability.

**Table 3:** Convergence validity of the ORCDi: Associations with rural-urban commuting area (RUCA) and social vulnerability index (SVI) domains.

Models	β (SE)	t-value	p-value	R^2^	AIC
RUCA	1.42 (0.01)	115.61	<0.0001	0.14	607925.3
SVI overall	13.97 (0.11)	126.71	<0.0001	0.16	605640.1
SVI socioeconomic	15.02 (0.11)	138.41	<0.0001	0.19	603078.0
SVI age & disability	15.74 (0.11)	146.68	<0.0001	0.21	601182.1
SVI minority status	−2.51 (0.12)	−20.94	<0.0001	0.01	619883.8
SVI Housing & Transportation	5.3 (0.12)	44.58	<0.0001	0.02	618357.2

**Notes:** Linear regression models were used to assess convergent validity of ORCDi against rural-urban commuting area (RUCA) and social vulnerability index (SVI) domains. β coefficients represent the change in ORCDi associated with a one-unit increase in each predictor. RUCA codes range from 1 (most urban) to 10 (most rural) and were treated as a continuous measure of the degree of rurality. SVI overall and domain scores range from 0 to 1, with higher values indicating greater social vulnerability. SE: Standard errors, t-values, and p-values indicate statistical significance. R^2^ reflects model explanatory power, and Akaike Information Criterion (AIC) indicates model fit.

The queen contiguity weights matric indicated that census tracts were well connected spatially, with an average of 6.19 neighboring tracts per unit, supporting stable estimation of spatial dependence (Table 4). Only 20 tracts lacked adjacent neighbors, suggesting minimal geographic isolation within the analytic dataset. Global Moran's I analysis demonstrated spatial autocorrelation in ORCDi (Moran's I = 0.72, z = 349.96, p < 0.0001), showed reflecting that ORCDi is clustered geographically rather than occurring independently across space. The magnitude of Moran's I and standardized deviate indicated substantial spatial dependence, reflecting concentrated patterns of high and low ORCD burden.

**Table 4:** Spatial weights characteristics and global Moran's I for ORCDi across U.S. census tracts.

Category	Metric	Value
Spatial weights structure	Number of census tracts	82,413
	Non-zero links	510,152
	Percentage non-zero weights	0.75%
	Average neighbors per tract	6.19
	Regions with no neighbors	20
	Weighting style	Row-standardized (W)
Global Moran's I test	Moran's I	0.71
	Expected value	−1.12 × 10^−5^
	Variance	4.18 × 10^−6^
	Z-score	346.19
	p-value	<0.0001
	Alternative hypothesis	Positive spatial autocorrelation

**Notes.** Global Moran's I was used to assess spatial autocorrelation of ORCDi across all census tracts using queen contiguity spatial weights. A positive Moran's I indicate clustering of similar index values. The large, standardized deviate and very small p-value reject spatial randomness, demonstrating significant positive spatial dependence in ORCDi values.

**Fig. 6:**
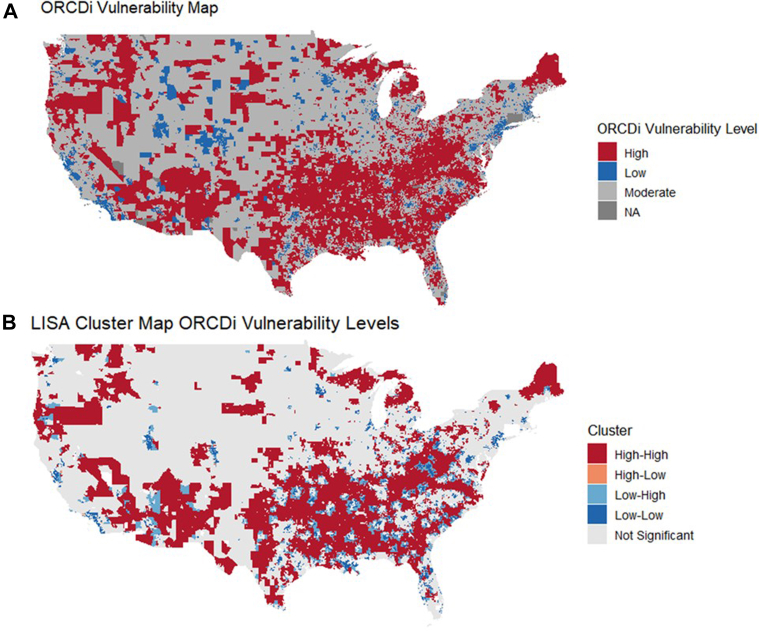
Geographic distribution and spatial clustering of the obesity-related chronic disease index (ORCDi) across U.S. census tracts. (A) Choropleth map illustrating ORCDi vulnerability levels (low, moderate, high) across the contiguous U.S. at the census tracts level. (B) Local indicators of spatial association (LISA) cluster map identifying significant spatial clusters or hotspot (high–high, low–low, high-low, low-high) of ORCDi values.

Fig. 6 illustrates the geographic distribution and spatial clustering or hotspots of the ORCDi across census tracts. High vulnerability tracts were densely concentrated across the Deep South, including Mississippi, Alabama, Louisiana, Arkansas, and eastern Texas, extending through the Mississippi Delta and central Appalachian region. Additional high-vulnerability clusters were observed in portions of Oklahoma and parts of the Southwest. In contrast, low ORCDi was more frequently observed in the Northeast corridor, upper Midwest, Rocky Mountain region, and along the Pacific Coast, particularly within metropolitan and suburban census tracts. Moderate vulnerability was broadly distributed across the Midwest, lower Great Plains, and interior Western states. The LISA cluster map (Fig. 6B) further demonstrated significant spatial dependence on ORCDi and statistical hotspots. Large contiguous high–high clusters indicated tracts with high ORCDi surrounded by similar high-burden neighbors, highlighting concentrated regional burden. Conversely, low–low clusters were primarily located in the Northeast, upper Midwest, and parts of the West Coast, reflecting areas of consistently lower multimorbidity. Scattered high-low and low-high tracts indicated localized spatial outliers, particularly at transitional boundaries between high- and low-burden regions.

Finally, to assess the robustness of ORCDi derivation and spatial inference, we conducted a comprehensive complete-case sensitivity analysis excluding census tracts with missing prevalence. Detailed results from the complete-case analysis were provided in the Supplementary Materials. PCA results were highly consistent with the primary imputed dataset, with comparable variance explained, stable factor loadings, and similar internal consistency measures (Supplementary Table S3). Spatial weights characteristics and Global Moran's I statistics also remained substantively similar (Supplementary Table S4), indicating persistent spatial autocorrelation. Furthermore, geographic vulnerability patterns in the choropleth map (Supplementary Fig. S1) and LISA cluster distributions (Supplementary Fig. S2) demonstrated nearly identical regional clustering. However, a proportion of census tracts in Florida contained missing prevalence and were excluded in the complete-case analysis. As a result, differences in spatial clustering were primarily observed within Florida, where local connectivity and cluster boundaries were unobserved.

## Discussion

This study developed and established the ORCDi, a novel composite metric designed to quantify ORCD burden at the U.S. census tracts level. The ORCDi demonstrated internal consistency, significant construct validity by the first principal component, and stable performance across training and testing datasets. Convergent validity was established through significant associations with social vulnerability and rural-urban classification, underscoring alignment with established structural determinants of health. Spatial analysis further demonstrated pronounced high–high clustering ORCDi across the Deep South, Mississippi Delta, central Appalachia, and parts of Texas and Oklahoma, underscoring persistent location-based inequities within historically underserved communities. Consistent with this pattern, Global Moran's I demonstrated positive spatial autocorrelation, highlighting the disproportionate geographic concentration of ORCD burden. Collectively, these findings establish ORCDi as a reliable, valid, and spatially sensitive index for monitoring ORCD burden and characterizing population-level health disparities across U.S. census tracts.

The construct validity of the ORCDi is supported by theoretical frameworks and empirical evidence linking obesity to multimorbidity, metabolic dysfunction, and social determinants of health. Consistent with prior literature, obesity has been linked to chronic disease through pathways such as systemic inflammation, insulin resistance, and hormonal dysregulation; however, our ecological and cross-sectional analysis does not directly test these mechanisms but rather identifies shared population-level patterns reflected in the dominant PC1.^31^ This clustering reflects the established concept of obesity-related multimorbidity, where a shared biological pathway leads to co-occurring cardiometabolic, inflammatory, and vascular conditions.^32^ The moderate loading of asthma aligns with growing evidence linking obesity to inflammatory disorders through bidirectional immune mechanisms.^33^ The significant association of ORCDi with socioeconomic vulnerability, disability burden, and rurality further reinforces its theoretical validity, consistent with the social determinants of health framework^34^ and spatial epidemiology theories that emphasize place-based inequities in chronic disease burden.^35^ Taken together, the clustering of obesity-related conditions and their alignment with structural vulnerability measures provide robust support for the construct validity and theoretical foundation of the ORCDi as a composite index.

The ORCDi demonstrated reliability and internal consistency, as evidenced by a Cronbach's alpha, a Guttman's lambda-6, and a mean inter-item correlation. These metrics significantly exceed the widely accepted thresholds for reliability, indicated expected internal consistency for population-level indices.^26^ The high alpha and lambda coefficients demonstrated that the nine chronic diseases shared latent construct rather than functioning independently. Significant item–total correlations and high discrimination indices demonstrated that each condition contributes meaningfully to the overall construct without redundancy, consistent with measurement expectations for a valid composite health index.^36^ Comparatively, the reliability statistics of ORCDi are similar to or higher than those reported for established population health indices such as the SVI domain and the RUCA categories.^37^^,^^38^ The high signal-to-noise ratio, minimal variance in inter-item correlations, and negligible standard error of Cronbach's alpha, together indicated measurement precision and stable reliability across diverse census tracts population. These findings align with measurement standards for population-level indices, where high internal consistency and low measurement error are essential for stability and interpretability.^39^^,^^40^

The convergent validity of the ORCDi was supported by its significant associations with established geographic and structural determinants. The index demonstrated a moderate association with RUCA, indicating that ORCD burden tends to intensify with increasing rurality and geographic isolation. This finding is consistent with prior research showing higher prevalence of cardiometabolic and inflammatory diseases, limited access to healthcare, and greater multimorbidity risk among residents of non-metropolitan areas.^41^ Moreover, RUCA is widely recognized as a standardized measure for capturing rural-urban health disparities in chronic disease surveillance.^15^^,^^42^ The observed variability in ORCDi across RUCA reinforces chronic disease burden is not spatially uniform, but context dependent.

The ORCDi also demonstrated alignment with SVI domain, particularly the age/disability and socioeconomic components. Census tracts with higher prevalence of older adults, disability, unemployment, and poverty tended to demonstrate substantially higher ORCDi. These findings are consistent with theories of structural vulnerability and syntomic models, which posit that chronic disease burden is clustered in communities characterized by social disadvantage, medical underservice, and compounding environmental stressors.^38^^,^^43^

Spatial clustering statistics further demonstrated a geographic coherence, with Global Moran's I, indicating spatial autocorrelation. High-risk spatial clusters were concentrated in the Deep South, Mississippi Delta, central Appalachia, and parts of Texas and Oklahoma-regions historically marked by high obesity prevalence, chronic disease multimorbidity, socioeconomic disadvantage, and geographic isolation.^44^ These patterns mirror well-established “stroke belt” and “diabetes belt” regions and align with prior spatial epidemiological studies identifying community-levels multimorbidity concentration.^45^ LISA analyses identified significant high–high and low–low clusters of ORCDi, revealing similar concentrations of elevated and reduced obesity-related multimorbidity across census tracts. These spatial clusters underscore persistent geographic inequities and support the need for targeted, place-based public health strategies.

To evaluate the robustness of the ORCDi, we conducted a complete-case sensitivity analyses excluding census tracts with missing values. The PCA structure remained stable, with comparable variance explained and consistent factor loadings across components, and internal consistency metrics showed minimal variation relative to the primary imputed analysis. Spatial connectivity metrics and Global Moran's I remained similar, indicating persistent and significant spatial autocorrelation. Choropleth and LISA patterns were largely consistent across analyses, with core high-burden clusters preserved; minor differences were observed primarily in Florida due to a proportion of excluded census tracts. Therefore, these results confirm the robustness of the ORCDi, with no material impact on index structure or spatial findings.

A key strength is its ability to enable fine-grained assessment of geographic and structural inequities. This index offers a rigorous, theory-driven framework supported by measurement performance and robust validation. Spatial autocorrelation and LISA analyses demonstrated significant community-level clustering, reinforcing its geographic coherence and utility for surveillance. However, this study has several limitations. The ecological design limits individual-level inference and may introduce aggregation bias. CDC PLACES estimates may be subject to model uncertainty in rural and low-population areas. Because CDC PLACES estimates are derived from model-based small-area estimation rather than direct individual-level measurements, they may be subject to uncertainty and potential bias, particularly in sparsely populated rural census tracts, and findings should be interpreted cautiously to avoid ecological fallacy. The ORCDi is derived from cross-sectional estimates and therefore does not capture disease severity, or duration. The index was internally evaluated but not externally validated against independent datasets or populations, which limits the generalizability. Future studies should assess the performance of this index across diverse geographic contexts and populations to confirm its broader applicability. Additionally, the index does not incorporate behavioral risk factors such as diet and physical activity, nor does it account for healthcare access or utilization, which may influence geographic variation in chronic disease burden. Findings reflect associative, population-level patterns rather than causal mechanisms, and future longitudinal validation using outcomes such as hospitalization and mortality would strengthen predictive and clinical relevance. Despite these limitations, the ORCDi is a robust and spatially sensitive index that identifies concentrated multimorbidity and aligns with social vulnerability and rurality, supporting scalable surveillance and equity-focused chronic disease planning.

In summary, the ORCDi provides a scalable, census tracts level tool for identifying communities with elevated ORCD burden and prioritizing areas for targeted intervention. Its alignment with social vulnerability and rural-urban patterns supports its application in community-levels prevention planning, resource allocation, and equity-focused public health strategies. The index can inform policymakers, health departments, and local stakeholders in designing geographically targeted programs, integrating findings into community health needs assessments and obesity-related chronic disease surveillance systems. By enabling fine-grained identification of high-burden areas, ORCDi facilitates data-driven decision-making aimed at reducing place-based disparities in chronic disease burden.

## Contributors

**MRA:** Conceptualization, Data curation, Formal analysis, Funding acquisition, Investigation, Methodology, Project administration, Resources, Software, Supervision, Validation, Visualization, Writing-original draft, Writing-review & editing. **FF:** Conceptualization, Methodology, Validation, Writing-review & editing, **XZ:** Conceptualization, Methodology, Validation, Writing-review & editing. **MRA**, **FF**, and **XZ** had full access to all the data in the study. MRA, and XZ have directly accessed and verified the underlying data reported in the manuscript. All authors have read and approved the final version of the manuscript and agree to be accountable for all aspects of the works. MRA made the final decision to submit the manuscript for publication.

## Data sharing statement

The datasets analyzed during the current study are publicly available. CDC PLACES data are available from the Centers for Disease Control and Prevention (CDC), Social Vulnerability Index data are available from the Agency for Toxic Substances and Disease Registry (ATSDR), and Rural–Urban Commuting Area classification data are available from the United States Department of Agriculture (USDA). Analytic code used for data processing and statistical analyses is available from the corresponding author upon reasonable request.

## Declaration of generative AI and AI-assisted technologies

During the preparation of this manuscript, the authors used ChatGPT (OpenAI) and Microsoft Copilot to assist with language refinement, technical writing support, and enhancement of clarity and readability. After using these tools, the authors manually reviewed, revised, and edited all generated content to ensure accuracy, originality, and compliance with scientific, ethical, and publication standards. The authors take full responsibility for the integrity, validity, and final content of the manuscript.

## Editor note

The Lancet Group takes a neutral position with respect to territorial claims in published maps and institutional affiliations.

## Declaration of interests

We declare no competing interests.
