# Supplementary material for: The obesity-related chronic disease index (ORCDi), a novel composite metric to quantify ORCD burden: an ecological study across U.S. census tracts
Source: Lancet Reg Health Am. 2026 Jul 1;61:101547. doi: 10.1016/j.lana.2026.101547 (PMC13355215; doi:10.1016/j.lana.2026.101547)
Supplement: Supplementary Materials [file mmc1.docx]

**The Obesity-Related Chronic Disease Index (ORCDi), a novel composite metric to quantify ORCD burden: An ecological study across U.S. Census Tracts**

**Supplementary Materials**

This document provides supplementary information that was not possible to include in the main text of the article because of constraints in terms of word counts and the number of tables and figures. The content of this supplemental material was submitted together with the main text of the article to the journal and peer reviewed.

**Table of Content**

[**Supplementary Methods** 2](#_Toc231720125)

[**Chronic disease definition** 2](#_Toc231720126)

[**Missing data handling** 3](#_Toc231720127)

[**Supplementary Results** 4](#_Toc231720128)

[**Supplementary Tables** 4](#_Toc231720129)

[**Supplementary Table S1.** Descriptive statistics, Kaiser–Meyer–Olkin (KMO) measures of sampling adequacy, Bartlett’s test of sphericity, and Jennrich test results for the primary (imputed) and complete-case analyses. 4](#_Toc231720130)

[**Supplementary Table S2:** Principal Component Loadings for Obesity-Related Chronic Disease Indicators and Variable Contributions (%) to the First Five Principal Components with Eigenvalues and Explained Variance of Principal Components. 5](#_Toc231720131)

[**Supplementary Table S3.** Complete-Case Sensitivity Analysis for obesity-related chronic disease index (ORCDi) Derivation. 6](#_Toc231720132)

[**Supplementary Table S4.** Comparison of Spatial Weights Structure and Global Moran’s I: Imputed vs Complete-Case Analyses. 7](#_Toc231720133)

[**Supplementary Figures** 8](#_Toc231720134)

[**Supplementary Figure S1**: Choropleth map illustrating ORCDi vulnerability levels (low, moderate, high) for complete case dataset across the contiguous United States at the census tract level. 8](#_Toc231720135)

[**Supplementary Figure S2**: Local Indicators of Spatial Association (LISA) cluster map identifying significant spatial clusters (high–high, low–low, high–low, low–high) of ORCDi for complete case dataset across U.S. census tracts. 9](#_Toc231720136)

# **Supplementary Methods**

## **Chronic disease definition**

Nine obesity-related chronic conditions were included in the derivation of the Obesity-Related Chronic Disease Index (ORCDi), based on age-adjusted prevalence estimates from the CDC PLACES 2024 modeling cycle (released 2025). All estimates were generated using multilevel regression and post-stratification applied to Behavioral Risk Factor Surveillance System (BRFSS) data.

The conditions included were:

- **Obesity:** Defined as body mass index (BMI) ≥30 kg/m² based on self-reported height and weight.
- **Diabetes:** Adults reporting a physician diagnosis of diabetes (excluding gestational diabetes).
- **High Blood Pressure (BPHIGH):** Adults ever told by a health professional that they had hypertension.
- **Coronary Heart Disease (CHD):** Adults reporting prior diagnosis of coronary heart disease.
- **Stroke:** Adults reporting prior diagnosis of stroke.
- **Chronic Obstructive Pulmonary Disease (COPD):** Adults reporting diagnosis of chronic bronchitis or emphysema.
- **Asthma:** Adults ever told by a health professional that they had asthma.
- **Arthritis:** Adults reporting diagnosis of arthritis, rheumatoid arthritis, gout, lupus, or fibromyalgia.
- **High Cholesterol (HIGHCHOL):** Adults told by a health professional that their blood cholesterol was high.

These conditions were selected based on established epidemiologic associations with obesity-related cardiometabolic and inflammatory disease burden and their relevance to population-level chronic disease surveillance.

## **Missing data handling**

Missing values were identified in the census tract-level prevalence estimates for high blood pressure and high cholesterol, affecting 5,077 of 83,522 census tracts (6.1%) for each variable (Supplementary Table S1). Because missingness was limited, confined to two indicators, and unlikely to substantially alter the underlying correlation structure, missing values were imputed using mean substitution. Specifically, each missing observation was replaced with the arithmetic mean of the corresponding non-missing prevalence estimates $x_{miss}=x=\frac{1}{n}\sum_{i=1}^{n} x_{i}$,

where $x_{i}$denotes the observed prevalence values and $n$represents the number of non-missing census tracts for the respective variable. This approach preserved complete geographic coverage and avoided the exclusion of census tracts from index derivation and spatial analyses. To evaluate the robustness of the imputation procedure, complete-case sensitivity analyses were performed by excluding census tracts with missing values. Principal component loadings, variance explained, internal consistency measures (Cronbach’s α and Guttman’s λ6), Kaiser–Meyer–Olkin statistics, Bartlett’s test of sphericity, Jennrich’s test, and spatial autocorrelation measures were recalculated and compared with the primary analyses.

# **Supplementary Results**

## **Supplementary Tables**

## **Supplementary Table S1.** Descriptive statistics, Kaiser–Meyer–Olkin (KMO) measures of sampling adequacy, Bartlett’s test of sphericity, and Jennrich test results for the primary (imputed) and complete-case analyses.

| **Characteristic** | **Primary Analysis (Imputed Data)** | | | **Complete-Case Analysis** | |
| --- | --- | --- | --- | --- | --- |
|  | **Missing**  **N (%)** | **Mean (SD) [Min, Max]**  **N = 83,522^1^** | **KMO-Measures** | **Mean (SD) [Min, Max]**  **N = 78,445*^1^*** | **KMO-Measures** |
| Stroke | 0 (0.0) | 3.7 (1.3) [0.2, 22.6] | 0.79 | 3.7 (1.3) [0.2, 22.6] | 0.79 |
| Arthritis | 0 (0.0) | 27.2 (6.8) [2.6, 59.1] | 0.82 | 27.1 (6.8) [2.6, 59.1] | 0.82 |
| Asthma | 0 (0.0) | 10.6 (1.4) [5.0, 20.7] | 0.73 | 10.7 (1.4) [5.0, 20.7] | 0.76 |
| Obesity | 0 (0.0) | 34.4 (7.2) [10.4, 64.4] | 0.77 | 34.6 (7.2) [10.4, 64.4] | 0.79 |
| COPD | 0 (0.0) | 7.5 (2.9) [0.7, 34.9] | 0.90 | 7.4 (2.9) [0.7, 34.9] | 0.91 |
| Coronary heart disease | 0 (0.0) | 7.0 (2.2) [0.4, 37.1] | 0.78 | 6.9 (2.1) [0.4, 37.1] | 0.71 |
| High blood pressure | 5,077 (6.1) | 33.8 (7.2) [4.1, 80.2] | 0.84 | 33.8 (7.4) [4.1, 80.2] | 0.84 |
| Diabetes | 0 (0.0) | 12.4 (3.8) [0.7, 45.7] | 0.80 | 12.4 (3.8) [0.7, 45.7] | 0.81 |
| High cholesterol | 5,077 (6.1) | 34.9 (4.5) [9.0, 58.9] | 0.79 | 34.9 (4.6) [9.0, 58.9] | 0.80 |
| Bartlett’s Test | 0 (0.0) | Overall MSA = 0.81, χ² = 1068915,  df = 36, p < 0.0001 | | Overall MSA = 0.82, χ² = 1057822, df = 36, p < 0.0001 | |
| Jennrich Test | 0 (0.0) | χ² = - 262.01, p >0.9 | | χ² = -618.7283, p >0.9 | |

**Note:** Values are presented as mean (SD) [minimum, maximum] for age-adjusted prevalence across 83,522 U.S. census tracts. Missing data are reported as the number and percentage of census tracts with unavailable values. Kaiser-Meyer-Olkin (KMO) statistics assess sampling adequacy for principal component analysis (PCA), whereas Bartlett’s test of sphericity evaluates the suitability of the correlation matrix for factor extraction. Jennrich’s test assesses the stability of the correlation structure across samples, with non-significant results indicating matrix equivalence. Abbreviations**:** MSA, measure of sampling adequacy; χ², chi-square statistic; df, degrees of freedom.

## **Supplementary Table S2:** Principal Component Loadings for Obesity-Related Chronic Disease Indicators and Variable Contributions (%) to the First Five Principal Components with Eigenvalues and Explained Variance of Principal Components.

| **Variable** | **Principal Component Loadings** | | | | | **Variable Contributions (%)** | | | | |
| --- | --- | --- | --- | --- | --- | --- | --- | --- | --- | --- |
|  | **PC1** | **PC2** | **PC3** | **PC4** | **PC5** | **PC1** | **PC2** | **PC3** | **PC4** | **PC5** |
| Stroke | 0.94 | 0.09 | -0.08 | -0.25 | 0.09 | 14.06 | 0.56 | 1.35 | 18.8 | 3.25 |
| Arthritis | 0.87 | -0.28 | 0.32 | 0.1 | -0.15 | 11.86 | 5.95 | 21.14 | 3.01 | 10.05 |
| Asthma | 0.57 | 0.71 | 0.32 | 0.1 | 0.26 | 5.05 | 37.58 | 20.61 | 2.73 | 28.13 |
| Obesity | 0.74 | 0.5 | -0.24 | 0.26 | -0.26 | 8.66 | 18.66 | 11.81 | 20.5 | 27.51 |
| COPD | 0.94 | 0.11 | 0.16 | -0.13 | -0.09 | 13.99 | 0.83 | 4.99 | 4.77 | 3.37 |
| Coronary heart disease | 0.91 | -0.27 | 0.16 | -0.19 | -0.11 | 13.07 | 5.56 | 5.28 | 11.1 | 5.19 |
| High blood pressure | 0.93 | -0.1 | -0.12 | 0.2 | 0.07 | 13.6 | 0.74 | 2.99 | 11.65 | 1.85 |
| Diabetes | 0.89 | 0.07 | -0.39 | -0.18 | 0.09 | 12.35 | 0.39 | 31.15 | 9.28 | 3.42 |
| High cholesterol | 0.68 | -0.63 | -0.06 | 0.25 | 0.2 | 7.37 | 29.73 | 0.68 | 18.17 | 17.22 |
| Eigenvalues | **6.35** | **1.33** | **0.49** | **0.34** | **0.24** | **70.52** | **14.75** | **5.45** | **3.74** | **2.65** |

**Note:** PC: principal components

## **Supplementary Table S3.** Complete-Case Sensitivity Analysis for obesity-related chronic disease index (ORCDi) Derivation.

| **Metric** | **Primary Analysis (Imputed Data)** | **Complete-Case Analysis** |
| --- | --- | --- |
| Number of census tracts | 83,522 | 78,445 |
| Variance explained (PC1, %) | 70.5 | 71.0 |
| Variance explained (PC2, %) | 14.8 | 14.4 |
| Cronbach’s α | 0.94 | 0.94 |
| Guttman’s λ6 | 0.97 | 0.97 |
| Global Moran’s I | 0.71 | 0.70 |
| Z-score | 346.19 | 337.38 |
| p-value | <0.0001 | <0.0001 |

**Note:** Principal component analysis was performed on standardized prevalence estimates of nine obesity-related chronic conditions. Loadings represent variable correlations with each principal component, and contributions (%) indicate the relative importance of each variable within a component. Eigenvalues and explained variance quantify the contribution of each principal component to the overall variability in the data. PC = principal component.

## **Supplementary Table S4.** Comparison of Spatial Weights Structure and Global Moran’s I: Imputed vs Complete-Case Analyses.

| **Category** | **Metric** | **Imputed Dataset** | **Complete-Case Dataset** |
| --- | --- | --- | --- |
| Spatial Weights Structure | Number of census tracts | 82,413 | 77,343 |
|  | Non-zero links | 510,152 | 478,444 |
|  | Percentage non-zero weights | 0.75% | 0.80% |
|  | Average neighbors per tract | 6.19 | 6.19 |
|  | Regions with no neighbors | 20 | 20 |
| Global Moran’s I Test | Weighting style | Row-standardized (W) | Row-standardized (W) |
|  | Moran’s I | 0.71 | 0.71 |
|  | Expected value | −1.12 × 10⁻⁵ | −1.29 × 10⁻⁵ |
|  | Variance | 4.18 × 10⁻⁶ | 4.46 × 10⁻⁶ |
|  | Z-score | 346.19 | 337.38 |
|  | p-value | <0.0001 | <0.0001 |
|  | Alternative hypothesis | Positive spatial autocorrelation | Positive spatial autocorrelation |

**Note:** Spatial autocorrelation was assessed using a first-order queen contiguity spatial weights matrix with row-standardized weights (W). Global Moran’s I statistics were calculated for both the primary imputed dataset and the complete-case dataset. Similar numbers of neighbors, spatial connectivity patterns, Moran’s I values, and significance levels across analyses indicate that missing-data imputation had minimal influence on the spatial structure or clustering of the ORCDi. Positive Moran’s I values indicate spatial clustering of similar index values. Abbreviations: W, row-standardized spatial weights; Moran’s I, Global Moran’s I statistic.

##

## **Supplementary Figures**


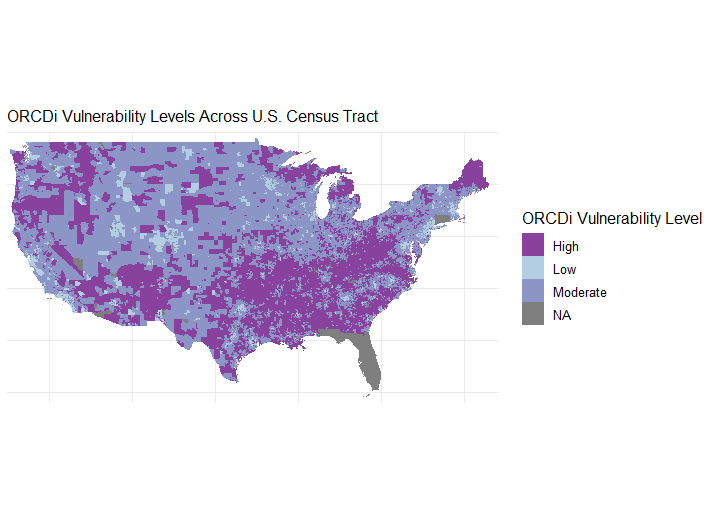


## **Supplementary Figure S1**: Choropleth map illustrating ORCDi vulnerability levels (low, moderate, high) for complete case dataset across the contiguous United States at the census tract level.


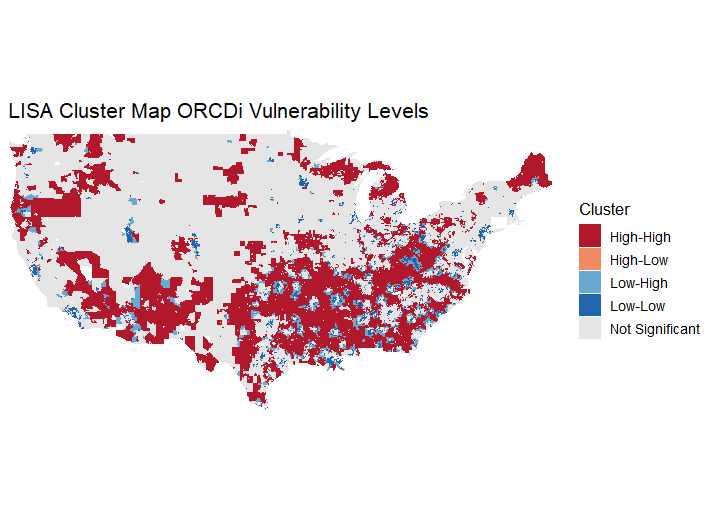


## **Supplementary Figure S2**: Local Indicators of Spatial Association (LISA) cluster map identifying significant spatial clusters (high–high, low–low, high–low, low–high) of ORCDi for complete case dataset across U.S. census tracts.
